# The Management of Chronic Invalids

**Published:** 1895-03

**Authors:** Geo. W. Christian

**Affiliations:** Houston, Texas


For Texas Medical Journal.

### THE MANAGEMENT OF CHRONIC INVALIDS.

BY GEO. W. CHRISTIAN, M. D., OF HOUSTON, TEXAS.

Read at Houston District Medical Society, December 31, 1894.

IF THE management of acute diseases taxes the knowledge and skill of the physician, he will often be put to more serious straits in handling the sub-acute and chronic troubles that will often confront him. Cases of chronic invalidism will be frequently met whose symptoms are so obscure and divers as to make it no easy task to locate the leading element which has brought the patient slowly from health to a state of utter worthlessness, to whining invalidism and misery.

The physician of acute observation and ripe experience will recognize in many patients, and especially among the more intelligent class of females suffering with the acute troubles so common to the female generation organs, a tendency to chronic invalidism, and should, by the exercise of a stronger will power than their own, endeavor to prevent such tendency. Nowhere in medicine can it be said with greater truth, "that an ounce of prevention is worth a pound of cure," than in this.

Chronic invalids will nearly all be found anæmic, have gone the rounds, too, from doctor to quack, from quack to fake, from fake to quack; have run the gauntlet of the various nostrums, tried all the tonics, magnetic healers, electric belts, Christian science fads, mineral waters, etc., all, too, to no purpose. Their list of complaints and symptoms are too long and numerous, too, for one man in an ordinary lifetime to acquire a knowledge of, though one redeeming feature nearly always exists in this class; that is, they can not remember all at once themselves, and will give it to you in broken doses as it were, but will generally insist, at each sitting, on commencing at the head of the list they have made out, for fear that you will not remember, or that they may have omitted something which, if neglected, might be the important link in the chain of their distress.

Our first efforts shold be devoted to securing the confidence of this class of patients, and to do this is probably the most trying and difficult part of our art. We must allow these patients to tell their tale in their own way, and consider with due deference their opinions. If we fail to do this in the outset, we will have lost ground that will be very hard ever to recover. After we have heard attentively what they have to say (and we can, by good generalship, generally help them through the long list), before giving an opinion, we should make a thorough examination of all vital organs, especially lungs, heart, stomach, bowels, liver, kidneys and generative organs, if they have any, and then, if we have gained their confidence, and can assure them of a cure in the end, our task will be a much easier one than might appear. The stronger we can make our first impressions, both mental and physical, the easier will be the road to a successful issue.

The nutrition of this class of patients is always under par, and we can make no substantial progress until we improve this. If the patient is of a constipated habit, treatment had better be begun with a *very active purge* and hot baths, followed by cold sponging and general massage.

The food should be light, nutritious, and easy of digestion, and in quantities to suit the digestive capacity; better keep the patient hungry than overdo this. A change of associations and surroundings, too, is always desirable. If no more can be done on this line than simply rearranging the room, better do so. The mind, too, should be cheerfully employed. Find out what will please and interest the patient most and direct their thoughts in that direction. Anything to occupy their attention, and divert their thoughts from themselves. Politics, art, literature, religion, dress, or something to divert them. Get them to do something in some line daily, and watch attentively their progress; encourage them by letting them see that you are interested in them. If bed-ridden, get them out, if only for a few moments, but be careful never to overtax their strength. Should you do this, you will have lost some of your influence over them.

Give whatever of drugs you will, but bear constantly in mind that no line will take the place of proper food, sunshine, fresh air, and cheerful employment. Is is by careful attention to the apparently insignificant details that we must succeed, if at all, in the relief of this class of patients.

I can better illustrate the application of the foregoing principles by rehearsing the histories of a few cases that have come under my personal observation:

Mrs. M., the wife of a prominent minister of this State, had been bed-ridden for two years. What first put her there, I am unable to tell. Her medical advisers had failed to get her out. She was sure she had some womb disease; her doctors assured her not. She had, in consequence, lost all confidence in their ability to cure her. I listened with attentive zeal to the history of her case. She made out a very strong one. After a careful examination, I found only a slight retroflexion of womb, certainly not of itself of sufficient import to account for the long period of invalidism. This was corrected, and patient persuaded out of bed; bowels vigorously moved, with directions for baths, sunshine, food and exercise. In ten days patient rode a distance of ten miles to my office, and five years afterwards, remained happy in the enjoyment of good health. Mrs. M. was about thirty-five years of age, and the mother of three or four children; has had one, to my knowledge, since something over a year after her recovery.

Mrs. G., about thirty years of age--no children—had been for ten years or more dragged around to different medical celebrities, one year of which was spent in this city, under some of your local celebrities. She was finally given over as entirely hopeless. She was quite a living skeleton, and withal a morphine habitue. Her bowels were either too loose or the reverse; appetite nil or ravenous. Had not been able to sit up for many months from weakness. The womb was found low down in the pelvis and fixed there from post-inflammatory action; hard to the touch, and nearly immovable. She was and had been liberally supplied with drugs, under which she had grown steadily worse. Her beef, wine, iron, malt, morphine, etc., were all confiscated. She was bathed daily with cold water, beginning with a wet towel and hurriedly given at first, and the plunge and shower given later, when she had gained sufficient strength to tolerate them. Was put upon a pure milk diet and kept on it alone for six weeks. The quantity, too, was regulated to suit the degestive capacity. Not more than eight ounces were given the first six hours, which was daily increased till one gallon daily was reached in the six weeks. The doors and windows were thrown wide open and kept so. Her body bared and exposed to the morning and evening sun for about one hour. Massage was given daily, commencing with about twenty minutes and gradually increasing till three hours a day was reached. Locally she had hot vaginal baths with vaginal and rectal massage. Her mind, at first, was put to rest, by not allowing any one to see her except her nurse and myself, and all literature denied. After four weeks she was allowed to see a few cheerful friends, and more gradually brought to reading and writing. In two months the patient was out of bed, in three months was walking three miles a day for exercise, in four months was in good flesh, round and rosy, looking well, feeling well, declaring that she was well, and after twelve years, she remains so. Now, I wish to emphasize that this case never took a dose of medicine after she fell into my hands, and is to-day a living monument to the power of cold water, sunshine, proper food and common sense.

Mrs. G. L., aged twenty-eight years; after five years of invalidism from imaginary kidney and liver disease, was by teaspoonful doses of water taken thrice daily, with proper bathing, feeding, exercise, and mental diversion, restored to health and usefulness.

One of the most trying experiences of my professional life was to get out of bed the wife of a well-to-do and over-indulgent husband. She had been an invalid for more than twenty years, and had not been out of bed, even to sit up, now for over two years; was very weak, and had lost about all the flesh she could spare. She clung to her invalidism with more than ordinary tenacity. After I had her red and rosy, fat and plump, she still declared herself no better, and would not stir. After getting rid of her husband, I decoyed her into a carriage, behind a lively team, and had her jolted, at a rapid pace, for ten miles over a rough road, and thereby broke the spell that bound her.

